# Taxonomy of the Genus *Scrobipalpa* Janse, 1951 (Lepidoptera: Gelechiidae) in Xinjiang of China †

**DOI:** 10.3390/insects17050491

**Published:** 2026-05-12

**Authors:** Zeqin Feng, Houhun Li, Oleksiy V. Bidzilya, Xiuying Zhang

**Affiliations:** 1 College of Life and Geographic Sciences, Key Laboratory of Biological Resources and Ecology of Pamirs Plateau in Xinjiang, Kashi University, Kashgar 844000, China; 15135131747@163.com; 2 College of Life Sciences, Nankai University, Tianjin 300071, China; 3 Institute for Evolutionary Ecology of the National Academy of Sciences of Ukraine, 37 Academician Lebediev Str., 03143 Kyiv, Ukraine; olexbid@gmail.com; 4 Staatliches Museum für Naturkunde Stuttgart, Rosenstein 1, D-70191 Stuttgart, Germany

**Keywords:** Gnorimoschemini, Microlepidoptera, new records, new species, Palaearctic region, taxonomy

## Abstract

This paper focuses on the taxonomy of the genus *Scrobipalpa* from Xinjiang, China. Four species—*Scrobipalpa apicidentata* Li, Bidzilya & Zhang sp. nov.; *S. latiuscula* Li, Bidzilya & Zhang sp. nov.; *S*. *subargenteonigra* Li, Bidzilya & Zhang sp. nov.; and *S. xinjiangensis* Li, Bidzilya & Zhang sp. nov.—are described as new. Seven species—*S. autonoma* Povolný, 1969; *S. concerna* Povolný, 1969; *S. dalibori* Lvovsky & Piskunov, 1989; *S. hannemanni* Povolný, 1966; *S. geomicta* (Meyrick, 1918); *S. picta* Povolný, 1969; and *S. zaitzevi* Piskunov, 1990—are newly recorded for the Chinese fauna. Images of adults and illustrations of genitalia of the new and newly recorded species are provided. A list of 48 *Scrobipalpa* species known in Xinjiang is included.

## Simple Summary

This study is based on the examination of specimens collected between 2023 and 2025 from Xinjiang, China. A total of 15 species of the genus *Scrobipalpa* are recognized during this study, which brings the number of the *Scrobipalpa* species now recognized in Xinjiang to 48. Of the 15 species newly added to the Xinjiang fauna, four are species new to science, seven are newly recorded species for China, and four are newly recorded species for Xinjiang. The results of the present study enrich the species resources, supplement relevant investigations in the arid and semi-arid areas, and provide basic data for the faunal distribution, the systematic evolution and the biodiversity of the genus *Scrobipalpa* in China.

## 1. Introduction

The genus *Scrobipalpa* was erected in 1951 by Janse, with *Gelechia heliopa* Lower, 1900 as the type species. It belongs to the tribe Gnorimoschemini, one of the most biodiverse groups in the family Gelechiidae (Bidzilya 2021) [1].

Povolný and Weismann (1958) revised the concept of *Scrobipalpa* [2], and Povolný established two monotypic genera, *Ilseopsis* Povolný, 1965 and *Ergasiola* Povolný, 1967 [3,4]. Povolný (2002) transferred the Palaearctic species previously included in *Scrobipalpa* to *Euscrobipalpa*, a subgenus of *Scrobipalpa* he had established in 1967, thereby treating the subgenus as a separate genus [5]. Owing to the diagnostic characteristics overlapping in both sexes among *Ilseopsis*, *Ergasiola* and *Euscrobipalpa*, this system has not been generally accepted by subsequent taxonomists, who still treat these three genera (sub-) as members of *Scrobipalpa*. To date, a total of 291 *Scrobipalpa* species have been recorded in the Palaearctic region (Bidzilya et al. 2022; Bidzilya 2023; Huemer and Özden 2024) [6,7,8], 10 species in the Nearctic Region (Lee et al. 2009) [9], 36 species in the Afrotropical Region, and six species in Australia (Bidzilya 2021) [1].

In China, the taxonomic study of the genus *Scrobipalpa* and even the tribe Gnorimoschemini started relatively late. Bidzilya and Li (2010) conducted the first systematic study on this genus in China, with a total of 56 species recorded [10]. Li and Bidzilya (2019) further described nine new species in science and newly recorded 13 species for the Chinese fauna. In the list of Li and Bidzilya (2019), *Scrobipalpa cryptica* Povolný, 1996 and *S. smithi* Povolný & Bradley, 1964 are removed from the list of *Scrobipalpa* of China due to misidentification [11]. In addition, Bidzilya et al. (2022) recorded the occurrence of *S. tenebrata* (Povolný, 2001) in China, and *S. zouhari* Povolný, 1984 is revised as a new synonym of *S. rebeli* (Preissecker, 1915), with both species previously recorded in China [6]. Prior to this study, a total of 76 species have been known in this country.

**Figure 1:**
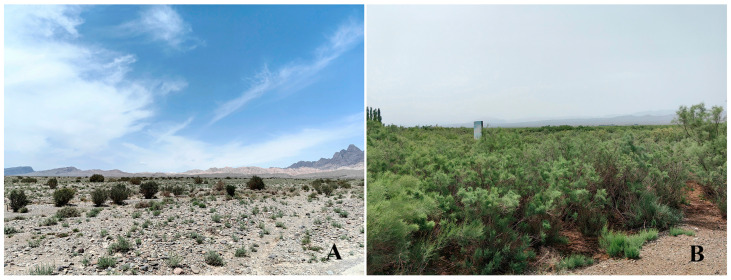
Arid and semi-arid environments in Xinjiang. (**A**). Gobi environment of Mingyaole Village, Shufu County, Kashi Prefecture. (**B**). Desert shrubland environment of Halajun Town, Artux City.

*Scrobipalpa* in China is mainly distributed in the arid and semi-arid regions (Figure 1), such as Xinjiang, Ningxia, Gansu, Inner Mongolia, Qinghai, Shaanxi and Tibet. Prior to this study, 33 species have been recorded to occur in Xinjiang (Li and Bidzilya 2019) [11].

The objectives of the present study are to describe four new species and to newly record seven species for the Chinese fauna based on the specimens collected in Xinjiang, to add four more species (*S. frugifera* Povolný, 1969, *S. grisea* Povolný, 1969, *S. subnitens* Povolný, 1969, *S. sattleri* Lvovsky & Piskunov, 1989) [11] to Xinjiang and to provide a list of all 48 *Scrobipalpa* species distributed in the Xinjiang Autonomous Region of China.

## 2. Materials and Methods

Specimens were collected by light traps. Genitalia dissection follows the methods introduced by Li [12]. Photographs of adults and genitalia were taken with a Leica M205A stereomicroscope (Leica Instruments (Singapore) Pte Ltd., 12 Teban Gardens Crescent, Jurong East Singapore) equipped with a Leica Application Suite X software (5.1.0.25593).

All the examined specimens, including the types of the new species, are deposited in the Insect Collection of Tianjin Natural History Museum, Tianjin (TJNHM) and the Insect Collection of Kashi University, Xinjiang (KSU), China.

The species accounts are arranged alphabetically. The descriptive terminology of genitalia structures generally follows Huemer and Karsholt (2010) [13].

## 3. Taxonomy

**Genus *Scrobipalpa* Janse, 1951**

*Scrobipalpa* Janse, 1951: 199. Type species: *Gelechia heliopa* Lower, 1900 [14].

*Ilseopsis* Povolný, 1965: 481. Type species: *Ilseopsis peterseni* Povolný, 1965 [3].

*Ergasiola* Povolný, 1967: 232. Type species: *Phthorimaea ergasima* Meyrick, 1916 [4].

*Euscrobipalpa* Povolný, 1967: 212. Type species: *Scrobipalpa grossa* Povolný, 1966 [4].

**Figure 2:**
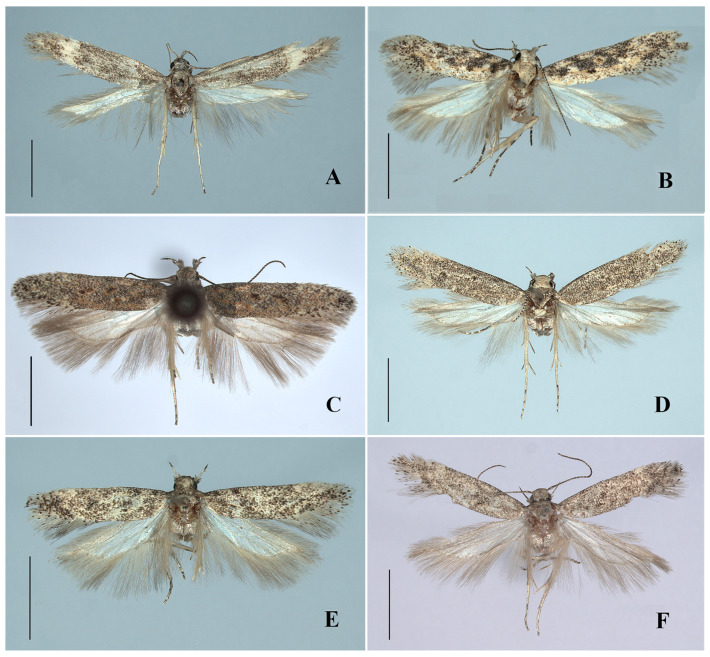
Adults of *Scrobipalpa* spp. (**A**). *S. apicidentata* sp. nov., ♂, holotype; (**B**). *S. autonoma* Povolný, 1969, ♂; (**C**). *S. concerna* Povolný, 1969, ♂; (**D**). *S. dalibori* Lvovsky & Piskunov, 1989, ♂; (**E**,**F**). *S. geomicta* (Meyrick, 1918), ♂. Scales = 2.0 mm.

***Scrobipalpa apicidentata* Li, Bidzilya & Zhang sp. nov.**
(Figure 2A and Figure 4A)
(LSID urn:lsid:zoobank.org:act:01D8FDC2-C4DB-4AE8-AD9E-A97E7ADFDF2B)

**Type material.** Holotype ♂, **CHINA, Xinjiang:** Kuoshitage Town, Pishan County (37.44° N, 78.14° E), 1601 m, 30.vii.2023, leg. A Gulzar and E Subinur, slide No. FZQ24419 (TJNHM).

Paratype: **Xinjiang:** 1 ♂, Xihexiu Town, Yecheng County, Kashi (36.98° N, 76.71° E), 2881.3 m, 14.vii.2025, leg. H.H. Li et al., slide No. FZQ25605 (KSU).

**Diagnosis.** The new species is defined externally by the forewing having a wide creamy white fascia at basal 3/5. It is similar to *S. krasnogorka* Bidzilya, Huemer & Šumpich, 2022 and *S. oleksiyella* Huemer & Karsholt, 2010 in the male genitalia, but differs in the sacculus, which is distinctly longer than the vincular process; the triangular vincular process is apical, with an inwardly curved denticle, and obliquely straight on the inner margin, and the gap between the sacculus and the vincular process is an inverted triangular shape. In *S. krasnogorka*, the sacculus is slightly longer than the vincular process, the subtriangular vincular process is narrowly rounded apically and is almost straight on the outer margin, and the gap between the sacculus and the vincular process is rectangular (Bidzilya, Huemer and Šumpich 2022: 27, Figures 104 and 105) [6]; in *S. oleksiyella*, the sacculus is slightly shorter than the vincular process, the irregularly shaped vincular process is narrowly rounded at the apex and its outer margin is concave below the apex, and the gap between the sacculus and the vincular process is irregularly trapezoidal (Huemer & Karsholt 2010: 78, Figure 31) [13].

**Description.** Adult (Figure 2A). Wingspan 14.5–15.0 mm.

*Head.* Vertex and frons greyish white. Antenna greyish brown. Labial palpus with segment 2 a greyish white, mixed with a few brown scales; segment 3 brown, almost as long as segment 2.

*Thorax.* Mesonotum and tegula greyish brown. Forewing brown, with a wide creamy white fascia at basal 3/5; fringe pale greyish brown. Hindwing pale greyish brown; fringe light brown.

*Abdomen.* Male genitalia (Figure 4A). Uncus elongate subrectangular, 1.5 times as long as wide, apex weakly concave medially, lateral corner obtusely rounded. Gnathos slender, slightly curved. Tegumen 2.5 times as long as middle width, with a deep and broad anteromedial emargination. Valva curved inward, wide at base, evenly slender from near base to before pointed apex, slightly exceeding apex of uncus. Sacculus ca. 1/4 length of valva, apex rounded, with a denticle directed inward. Vinculum with a deep V-shaped posteromedial emargination; vincular process triangular, shorter than sacculus, with a sharp denticle slightly curved outwardly, gap between vincular process and sacculus inverted triangular, posteriorly approximately twice width of sacculus. Saccus broad at base, narrowed to pointed apex, extending slightly beyond pedunculus apically. Phallus straight; caecum inflated, 1/2 length of phallus; apical arm short, curved, ca. 1/8 length of phallus.

Female. Unknown.

**Distribution.** China (Xinjiang).

**Etymology.** The specific epithet is derived from the Latin *apic* (apex) and *dentatus* (of denticle), referring to the apical teeth at apices of the sacculus and the vincular process.

***Scrobipalpa autonoma* Povolný, 1969**
(Figure 2B, Figures 4B and 6A)

*Scrobipalpa autonoma* Povolný, 1969a: 20. TL: Mongolia. TD: HNHM [15].

*Euscrobipalpa autonoma*: Povolný, 2002: 33 [5].

**Material examined. CHINA, Xinjiang:** 1 ♂, Taer Town, Akto County (37.80° N, 76.89° E), 2184 m, 18.vii.2024, leg. XY Zhang, SL Zhang and ZQ Feng, slide No. FZQ24298 (KSU); 1♂3♀, Nushidun Village, Maeryang Town, Taxkorgan County, Kashi (37.34° N, 75.84° E) (TJNHM), 2079 m, 28.vii.2024, leg. M. Nuriye and LX Liu, slide Nos. FZQ25103♂, FZQ25062♀, FZQ25091♀, FZQ25189♀; 1 ♂, Mazhaer Village, Taxkorgan County, Kashi (37.15° N, 75.45° E), 3727 m, 1.viii.2024, leg. M. Nuriye and LX Liu, slide No. FZQ24107 (KSU); 3 ♂, Mushi Town, Shufu County, Kashi City (39.58° N, 75.54° E), 1736.5 m, 6.vi.2025, leg. YQ Zhai and XY Zhang, slide Nos. FZQ25368, FZQ25369, FZQ25370 (TJNHM); 1 ♂, Kan-Areli Village, Halajun Town, Artux City, Kizilsu Kirgiz Autonomous Prefecture (40.14° N, 76.78° E), 1626.2 m, 25.v.2025, leg. ZQ Feng and C Cheng, slide No. FZQ25298 (KSU); 1 ♂, Kangshiweier Forest Farm, Wuqia County, Kizilsu Kirgiz Autonomous Prefecture (40.14° N, 76.78° E), 2295.5 m, 6.vi.2025, leg. XY Zhang and SL Zhang, slide No. FZQ25367 (KSU); 1 ♂, Yeketireke Village, Wuqia County, Kizilsu Kirgiz Autonomous Prefecture (39.72° N, 75.21° E), 2115.6 m, 7.vi.2025, leg. SL Zhang and XH Liu, slide No. FZQ25365 (TJNHM); 2 ♂, Eastern Tamarix Forest, Halajun Village, Artux City, Kizilsu Kirgiz Autonomous Prefecture (40.18° N, 76.85° E), 1632.5 m, 25.v.2025, leg. YQ Zhai and C Cheng, slide Nos. FZQ25297, FZQ25317 (TJNHM); 1 ♂, Riverside, Langru Town, Hetian County (36.90° N, 79.40° E), 1800 m, 3.viii.2024, leg. HH Li and Aishanjiang, slide No. ZYQ25298 (TJNHM).

**Diagnosis.** Adult (Figure 2B). Wingspan 12.5–13.0 mm. *Scrobipalpa autonoma* is distinguished by the forewing with large ill-defined dark brown patches from the base to the distal 1/4 between the costal margin and the fold. The male genitalia are distinguished by the apex of the trapezoidal uncus being shallowly concave at middle, the tegumen with a broad semicircular anteromedial emargination, the valva bent inward and slightly exceeding the apex of the uncus, the vinculum with a deep U-shaped posteromedial emargination and a subtriangular vincular process (Figure 4B). The female genitalia are distinguished by the subgenital plate covered with microspines from the middle length of the inner margin to the base of the apophyses anteriores, the trapezoidal ventromedial depression divided by a triangular anteromedial incision into subovate lobes that slightly exceed the anterior margin of the sternite VIII, and the globular corpus bursae (Figure 6A).

**Distribution.** China (Xinjiang), Mongolia [5].

**Note.** This species is newly recorded in China.

***Scrobipalpa concerna* Povolný, 1969**
(Figure 2C and Figure 4C)

*Scrobipalpa concerna* Povolný, 1969a: 21. TL: Mongolia. TD: HNHM [15].

*Euscrobipalpa concerna* Povolný, 2002: 37 [5].

*Scrobipalpa concerna uzbeka* Falkovitsh & Bidzilya, 2006: 70 [16].

**Material examined. CHINA, Xinjiang:** 3♂, Xindi Timber Inspection Station, Jimusaer County, Changji Hui Autonomous Prefecture (43.83° N, 88.94° E), 1595 m, 9.viii.2025, leg. A Gulzar and QY Min, slide Nos. ZYQ25563, ZYQ25566, ZYQ25570 (TJNHM and KSU).

**Diagnosis.** Adult (Figure 2C). Wingspan 10.0–11.0 mm. *Scrobipalpa concerna* is distinguished by the brown forewing mixed with a few creamy yellow and ochreous yellow scales, and with black spots surrounded by ocherous yellow scales. The male genitalia are distinguished by the subquadrate uncus with apex weakly concave at middle, the apically inflated valva slightly curved inward and not extending beyond the apex of the uncus, and the vinculum with a V-shaped posterior emargination (Figure 4C).

*Scrobipalpa concerna* is similar to *S. dagmaris* Povolný, 1987, but differs in the forewing, with smaller black spots surrounded by ochreous scales, in the broad subquadrate uncus, the valva dilated apically, the sacculus rounded at apex, and the stouter vincular process apically reaching below the apex of the sacculus. In *S. dagmaris*, the forewing has larger black spots without distinct ochreous scales surrounding them; in the male genitalia the uncus is elongate trapezoidal, the valva is dilated before the apex, the sacculus is acute at apex, and the slender vincular process slightly exceeds the apex of the sacculus apically (Povolný 2002: 38, Figures 4 and 241) [5].

**Distribution.** China (Xinjiang), Mongolia, Uzbekistan [5,16].

**Note.** This species is newly recorded in China.

***Scrobipalpa dalibori* Lvovsky & Piskunov, 1989**
(Figure 2D and Figure 4D)

*Scrobipalpa dalibori* Lvovsky & Piskunov, 1989: 537. TL: Mongolia. TD: ZIRAS (= ZIN) [17].

*Euscrobipalpa cultrata* Lvovsky & Piskunov, 1989, Povolný, 2002: 38 [5].

**Material examined. CHINA, Xinjiang:** 1♂, Maeryang Town, Taxkorgan Tajik Autonomous County, Kashi (37.31° N, 75.85° E), 2656 m, 7.viii.2024, leg. Nuriye, LX Liu and YQ Zhai, slide No. FZQ25067 (KSU); 3♂, Heiziwei Town, Wuqia County, Kizilsu Kirgiz Autonomous Prefecture (37.72° N, 75.21° E), 2067.4 m, 25.v.2025, leg. XY Zhang, YQ Zhai and ZQ Feng, slide Nos. ZYQ25347, ZYQ25350 (TJNHM); 1♂, Kangshiweir Forest Farm, Wuqia County, Kizilsu Kirghiz Autonomous Prefecture (39.73° N, 75.39° E), 2126.11 m, 9.vi.2025, leg. Shuli Zhang and Xiuying Zhang, slide No. ZYQ25362 (TJNHM).

**Diagnosis.** Adult (Figure 2D). Wingspan 12.5–13.0 mm. *Scrobipalpa dalibori* is similar to *S. krasilnikovae* Piskunov, 1990 superficially. It differs from the latter in the forewing having indistinct black spots, the male genitalia in the valva extending beyond the apex of the uncus, the vincular process reaching the apex of the sacculus, and the much stouter phallus (Figure 4D). In *S. krasilnikovae*, the forewing has distinct black spots, the valva does not reach the apex of the uncus, the vincular process reaches far below the apex of the sacculus, and the phallus is rather slender in the male genitalia (Povolný 2002: 52, Figure 278) [5].

The male genitalia of *S. dalibori* are also similar to those of *S. vartianorum* (Povolný, 1968) but can be distinguished by the uncus being obtuse at the apex, the sacculus uniformly wide to the rounded apex, and the sinuate vincular process bent outward distally (Figure 4D). In *S. vartianorum*, the uncus is slightly concave at the apex medially, the sacculus is slightly narrowed distally, and the vincular process extends obliquely inward (Povolný 1968: 16, Figure 66) [18].

**Distribution.** China (Xinjiang), Mongolia [17].

**Note.** This species is newly recorded in China.

***Scrobipalpa geomicta* (Meyrick, 1918)**
(Figure 2E,F, Figures 4E and 6B)

*Phthorimaea geomicta* Meyrick, 1918: 18. TL: South Africa. TD: TMSA [19].

*Phthorimaea vicaria* Meyrick, 1921: 74 [20].

*Scrobipalpa geomicta*: Janse 1951: 208 [14].

*Scrobipalpa vicaria*: Janse 1951: 220 [14].

*Scrobipalpa tineiformis* Povolný, 1967: 230 [4].

*Scrobipalpa geomicta*: Bidzilya 2021: 36 [1].

**Material examined. CHINA, Xinjiang:** 13♂1♀, Mushi Town, Shufu County, Kashi (39.58° N, 75.54° E), 1736.5 m, 6.vi.2025, leg. YQ Zhai and XY Zhang, slide Nos. FZQ25043♂, FZQ25049♂, FZQ25050♂, ZYQ25336♂, ZYQ25338♂, ZYQ25340♂, FZQ25344♂, FZQ25348♂, FZQ25349♂, FZQ25353♂, FZQ25360♂, ZYQ25348♂, FZQ25350♂, FZQ25114♀ (TJNHM and KSU); 3♂2♀, Honghai Scenic Area, Bachu County (39.77° N, 78.29° E), 1127.1 m, 16.vi.2024, leg. XY Zhang, GH Chen and SL Zhang, slide Nos. FZQ25205♂, FZQ25153♂, FZQ24439♂, FZQ25015♀, FZQ25019♀ (TJNHM); 1♂ Xinju, Tumushuke City (39.92° N, 79.35° E), 1043 m, 13.viii.2024, leg. GH Chen, QY Min and X Liu, slide No. FZQ25148♂ (KSU); 1♂1♀, Saikai Kule, Yuli County (39.79° N, 76.40° E), 877 m, 7.viii.2023, leg. A Gulzar and E Subinur, slide Nos. ZYQ25113♂, FZQ24403♀(TJNHM); 3 ♂, Gedaliang Town, Artux City, Kizilsu Kirgiz Autonomous Prefecture (39.79° N, 76.40° E), 1213 m, 6.v.2024, leg. A Gulzar and LX Liu, slide Nos. FZQ25171, FZQ24222, FZQ24429 (TJNHM).

**Diagnosis.** Adult (Figure 2E,F). Wingspan 8.0–12.0 mm. *Scrobipalpa geomicta* (Meyrick, 1918) is characterized in the male genitalia by the gradually narrowed uncus longer than its proximal width, the sacculus distally narrowing to the inwardly pointed apex, the vinculum with a large ovate posterior emargination, the vincular process extending to about 3/4 the length of the sacculus, and the broad saccus straight at the apex (Figure 4E). The female genitalia are distinguished by the teardrop-shaped anteromedial depression with distinctly edged lateral edges, the boundary between the ductus bursae and the corpus bursae being indistinct, and the signum slightly curved (Figure 6B).

**Distribution.** China (Xinjiang), Spain, Greece, Mongolia, W. India, Ethiopia, South Africa and Namibia (Bidzilya 2021: 38) [1], Malta, Morocco, Libya, Cyprus, Saudi Arabia, Iran, Pakistan (Huemer and Karsholt 2010: 163) [13].

**Note.** This species is newly recorded in China. By comparison we found the forewings of the individuals collected in Xinjiang have ill-defined black dots and are lighter in colour than those reported by Bidzilya (2021) [1] in the Afrotropical region.

**Figure 3:**
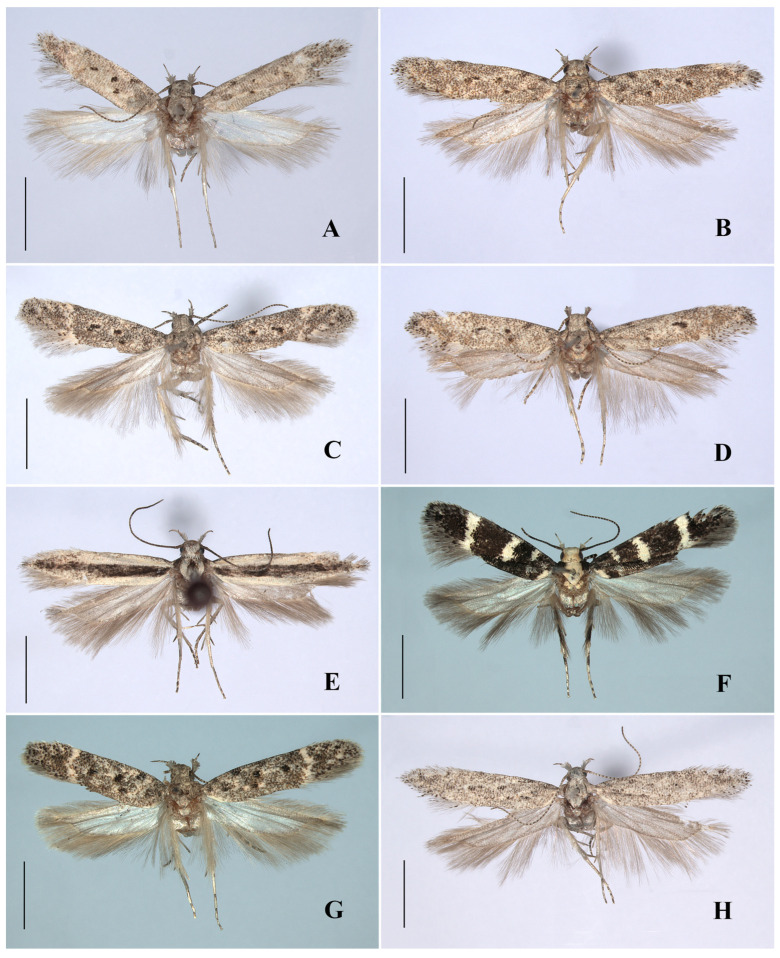
Adults of *Scrobipalpa* spp. (**A**). *S. hannemanni* Povolný, 1966, ♂; (**B**). *S. latiuscula* sp. nov., ♂, holotype; (**C**,**D**). *S. latiuscula* sp. nov., ♂, paratype; (**E**). *S. picta* Povolný, 1969, ♂; (**F**). *S. subargenteonigra* sp. nov., ♂, holotype; (**G**). *S. xinjiangensis* sp. nov., ♂, holotype; (**H**). *S. zaitzevi* Piskunov, 1990, ♂. Scales = 2.0 mm.

**Figure 4:**
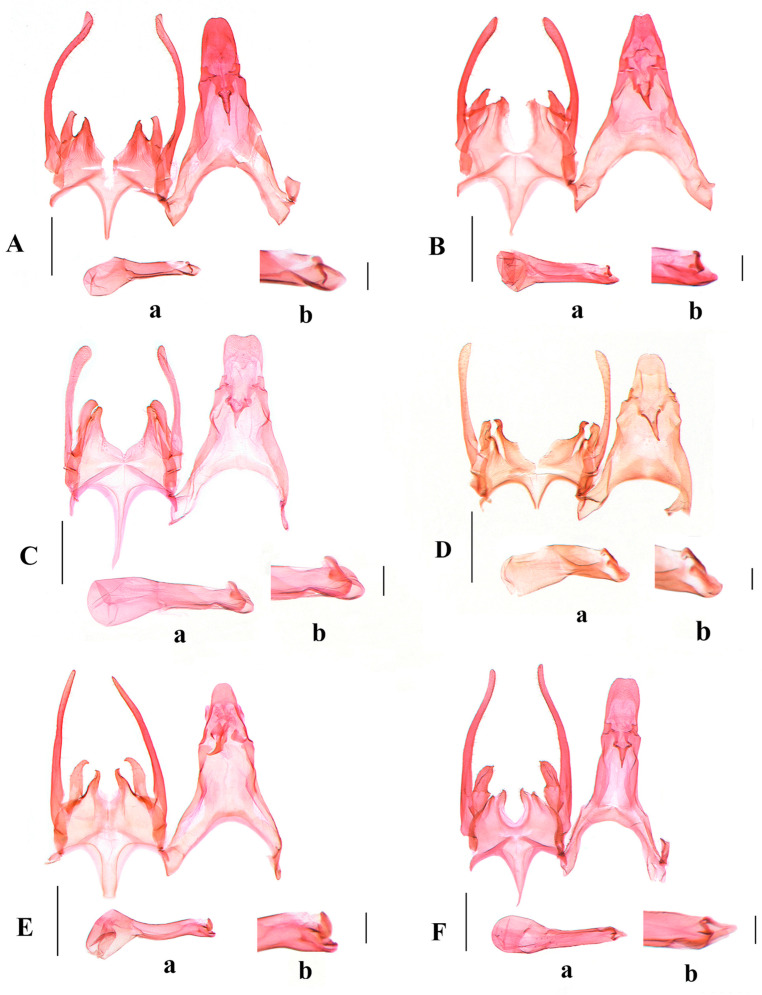
Male genitalia of *Scrobipalpa* spp. (**A**). *S. apicidentata* sp. nov., slide No. FZQ24419, holotype; (**B**). *S. autonoma* Povolný, 1969, slide No. FZQ24298; (**C**). *S. concerna* Povolný,1969, slide No. ZYQ25566; (**D**). *S. dalibori* Lvovsky & Piskunov, 1989, slide No. FZQ25067; (**E**). *S. geomicta* (Meyrick, 1918), slide No. FZQ25360; (**F**). *S. hannemanni* Povolný, 1966, slide No. FZQ25496. (a. phallus, b. enlarged apical arm). Scales: a = 0.25 mm, b = 0.05 mm.

***Scrobipalpa hannemanni* Povolný, 1966**
(Figure 3A, Figure 4F and 6C)

*Scrobipalpa hannemanni* Povolný, 1966a: 402. TL: Croatia. TD: MfN (= ZMB) [21].

*Scrobipalpa hannemanni furva* Povolný, 1969a: 12 [15].

*Euscrobipalpa hannemanni hannemanni* (Povolný, 1966), Povolný, 2002: 46 [5].

*Euscrobipalpa hannemanni gamanthi* Falkovitsh & Bidzilya, 2006: 77 [16].

*Scrobipalpa hannemanni*: Huemer & Karsholt, 2010: 168 [13].

**Material examined. CHINA, Xinjiang:** 13♂2♀, Near Kizilia Grand Canyon, Kuche City (42.10° N, 83.04° E), 1487 m, 28.vii.2025, leg. SX Wang, Aishanjiang and QY Min, slide Nos. FZQ25488♂, FZQ25493♂, FZQ25496♂, FZQ25497♂, FZQ25548♂, FZQ25582♂, FZQ25670♂, FZQ25673♂, FZQ25674♂, ZYQ25499♂, ZYQ25504♂, FZQ25546♀ (TJNHM and KSU).

**Diagnosis.** Adult (Figure 3A). Wingspan 9.0–9.5 mm. *Scrobipalpa hannemanni* is similar to *S. eremica* Povolný, 1967 superficially, but can be separated by the genital features. The male genitalia differ in the inwardly curved valva not inflated apically and far exceeding the apex of the uncus, the sacculus of equal width, and the vinculum with a V-shaped posteromedial emargination (Figure 4F). The female genitalia differ in the subgenital plate, with foamy sculptures from the posterior 1/4 of the inner margin to the base of the anterior apophyses, and the signum with spines (Figure 6C). In *S. eremica*, the straight valva is dilated at the apex that does not exceed the apex of the uncus, the sacculus is widened beyond the middle, and the vinculum has a U-shaped posteromedial emargination in the male genitalia; the subgenital plate only possesses foamy sculptures at the base of the anterior apophyses, and the signum lacks spines in the female genitalia (Povolný 2002: 41, Figures 17, 289 and 762) [5].

**Distribution.** China (Xinjiang), Mongolia (Povolný 2002) [5], Croatia (Povolný 1966a) [21], Uzbekistan (Falkovitsh and Bidzilya 2006) [16], Russia (Lower Volga region, S Ural, Zabaikalskiy krai) (Bidzilya 2009) [22]; Ponomarenko 2019 [23]).

Subspecies *S. h. furva* Povolný, 1969 in Mongolia [5], and subspecies *S. h. gamanthi* Falkovitsh & Bidzilya, 2006 in Uzbekistan [16].

**Note.** This species is newly recorded in China.

**Figure 5:**
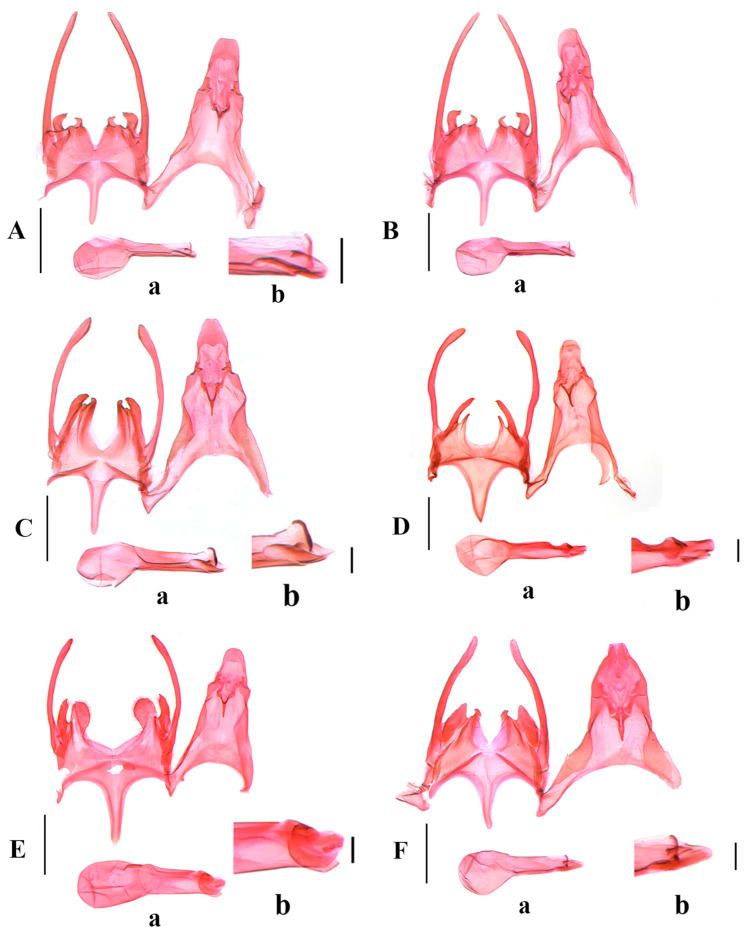
Male genitalia of *Scrobipalpa* spp. (**A**). *S. latiuscula* sp. nov., slide No. FZQ25685, holotype; (**B**). *S. latiuscula* sp. nov., slide No. FZQ25597, paratype; (**C**). *S. picta* Povolný, 1969, slide No. FZQ25465; (**D**). *S. subargenteonigra* sp. nov., slide No. FZQ25383, holotype; (**E**). *S. xinjiangensis* sp. nov., slide No. FZQ25377, holotype; (**F**). *S. zaitzevi* Piskunov, 1990, slide No. ZYQ25679. (a. phallus, b. enlarged apical arm). Scales: a = 0.25 mm, b = 0.05 mm.

**Figure 6:**
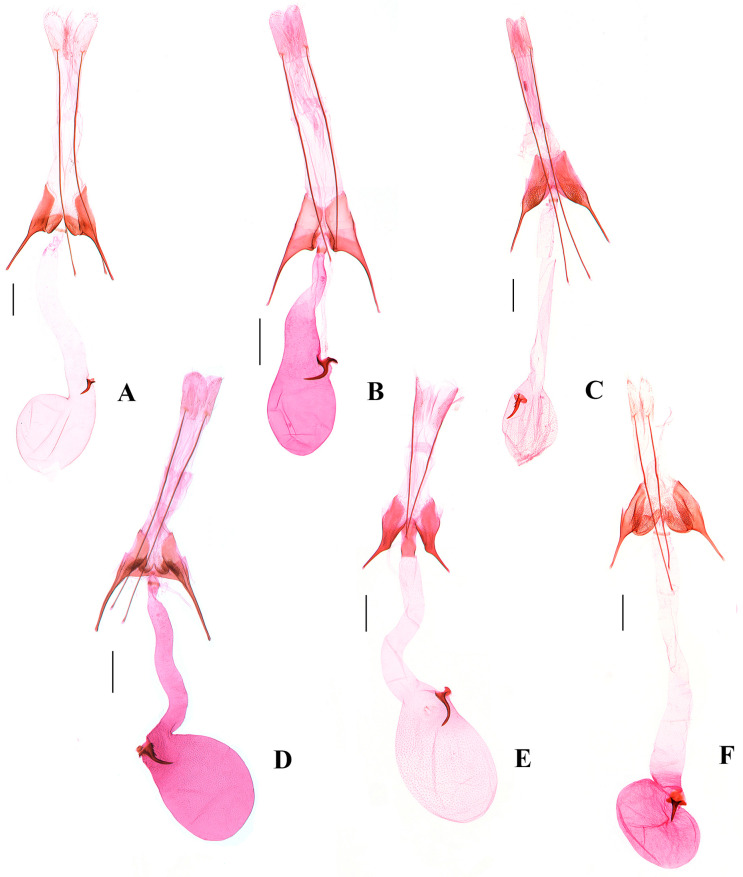
Female genitalia of *Scrobipalpa* spp. (**A**). *S. autonoma* Povolný, 1969, slide No. FZQ25062; (**B**). *S. geomicta* (Meyrick, 1918), slide No. FZQ25114; (**C**). *S. hannemanni* Povolný, 1966, slide No. FZQ25546; (**D**). *S. latiuscula* sp. nov., slide No. FZQ25762, paratype; (**E**). *S. subargenteonigra* sp. nov., slide No. ZYQ25132, paratype; (**F**). *S. xinjiangensis* sp. nov., slide No. FZQ25382, paratype. Scales = 0.25 mm.

***Scrobipalpa latiuscula* Li, Bidzilya & Zhang sp. nov.**
(Figure 3B–D, Figure 5A,B and Figure 6D)
(LSIDurn:lsid:zoobank.org:act:C3D7BFB1-DFAF-4582-8B1F-7E94F5877CB9)

**Type material.** Holotype ♂, **CHINA, Xinjiang:** Halajun Township, Artux City, Kizilsu Kirghiz Autonomous Prefecture (40.13° N, 76.77° E), 1585 m, 21.vii.2025, leg. XY Zhang, XH Liu and ZQ Feng, gen. slide no. FZQ25685 (TJNHM).

Paratypes: **Xinjiang:** 3♂, same data as holotype, slide Nos. FZQ25513, FZQ25522, FZQ25530; 18♂3♀, Gedaliang Township, Artux City, Kizilsu Kirghiz Autonomous Prefecture (39.80° N, 76.61° E), 1165 m, 20.vii.2025, leg. XY Zhang, YQ Zhai and XH Liu, slide Nos. FZQ25509♂, FZQ25570♂, FZQ25597♂, FZQ25616♂, FZQ25618♂, FZQ25625♂, FZQ25635♂, FZQ25641♂, FZQ25650♂, FZQ25656♂, FZQ25657♂, FZQ25661♂, FZQ25672♂, FZQ25675♂, FZQ25677♂, FZQ25690♂, FZQ25761♀, FZQ25762♀; 1 ♂, South of Halatuore Village, Jinghe County, Bortala Mongol Autonomous Prefecture (44.66° N, 82.86° E), 263 m, 4.viii.2025, leg. HH Li and SX Wang et al., slide No. FZQ25460 (TJNHM and KSU); 1 ♂, Mutetar Desert, Jinghe County, Bortala Mongol Autonomous Prefecture (44.62° N, 83.56° E), 310 m, 5.viii.2025, leg. HH Li and SX Wang et al., slide No. FZQ25510 (TJNHM).

**Diagnosis.** The new species is similar to *S. xinjiangensis* sp. nov. superficially. It can be distinguished in the male genitalia by the sacculus with the apex produced inward and forming an angle with the inner margin, the vincular process shorter and narrower than the sacculus, and the phallus dilated basally; in the female genitalia it can be distinguished by the hourglass-shaped ventromedial depression with its lobes not exceeding the anterior margin of the subgenital plate. In *S. xinjiangensis*, the sacculus is not produced inward apically, the broadly dilated vincular process is as long as and more than twice as wide as the sacculus, and the phallus is not distinctly dilated basally; in the female genitalia, the ventromedial depression is trapezoidal, and its lobes exceed the anterior margin of the subgenital plate.

**Description. Adult** (Figure 3B–D). Wingspan 9.0–9.5 mm.

*Head.* Vertex and frons creamy white, mixed with greyish brown tipped scales. Antenna with scape creamy white finely annulated with dark brown; flagellum creamy white alternating with dark brown. Labial palpus off-white; segment 2 with a few dark brown scales on outer side, tipped with dark brown on ventral surface; segment 3 with dark brown on ventral surface except at apex, with a dark brown ring at base, dark brown from beyond middle to before apex on dorsal surface.

*Thorax.* Mesonotum and tegula creamy, mixed with greyish brown scales. Forewing creamy white, mixed with light brown to fuscous scales; indistinct black spot at base and at 1/5 of costal margin; distinct black spot at basal 1/3 and 3/5 of wing, surrounded by yellowish brown scales, fold with indistinct black spot at base, with more distinct spot before basal 1/4 and before middle, respectively; creamy white subterminal fascia extending zigzag from 3/4 of costal margin obliquely inward to tornus; fringe yellowish grey, tipped with dark brown. Hindwing white, slightly tinged with grey; fringe yellowish grey.

*Abdomen.* Male genitalia (Figure 5A,B). Uncus subrectangular, twice as long as medial width, slightly narrowed to obtuse apex. Gnathos long, slightly curved distally. Tegumen 2.5 times as long as middle width, with a deep semicircular anterior emargination. Valva uniformly slender, apex narrowly rounded, extending far beyond apex of uncus. Sacculus ca. 1/6 length of valva, parallel sided to apex; apex extending inward, forming a right or obtuse angle with inner margin; vinculum with a V-shaped posterior emargination; vincular process slightly shorter than sacculus, broad at base, gradually narrowed to before apex; apex pointed and strongly curved outward, forming a right angle with inner margin. Saccus slightly broadened at base, uniformly slender from near base to before pointed apex, slightly extending beyond pedunculus. Phallus straight; caecum inflated, ca. 2/5 length of phallus; apical arm slender, slightly curved distally, ca. 1/9 length of phallus.

Female genitalia (Figure 6D). Papilla analis subovate, with sparse short setae. Apophyses posteriores ca. four times longer than apophyses anteriores. Sternite VIII trapezoidal. Subgenital plate 1/3 width of sternite VIII in middle, bearing foamy sculptures from middle of inner margin to base of apophyses anteriores. Ventromedial depression hourglass-shaped, covered with fine microspines, divided by triangular anteromedial incision to 1/3 of its length, forming two large subovate lobes, each lobe with foamy sculptures, not extending beyond anterior margin of sternite VIII. Colliculum short, ring-shaped. Ductus bursae of uniform width from posterior 1/6 to corpus bursae, curved. Corpus bursae pyriform; signum situated near entrance of corpus bursae, distal hook weakly curved.

**Distribution.** China (Xinjiang).

**Etymology.** The specific epithet is derived from the Latin *latiusculus*, referring to the relatively wide gap between the sacculus and the vincular process in the male genitalia.

**Variations.** Adults of the new species exhibit considerable variations. Forewings in some individuals lack the distinct zigzag creamy-white subterminal fascia, and have indistinct black spots.

***Scrobipalpa picta* Povolný, 1969**
(Figure 3E and Figure 5C)

*Scrobipalpa (Euscrobipalpa) picta* Povolný, 1969b: 375. TL: Afghanistan. TD: SMNK (= LNK) [24].

*Euscrobipalpa picta*: Povolný, 2002: 62 [5].

**Material examined. CHINA, Xinjiang:** 1 ♂, West of Continental Bridge Oil Products Company, Kuitun City, Ili Kazakh Autonomous Prefecture (44.41° N, 84.87° E), 527 m, 6.viii.2025, leg. A Gulzar and QY Min, slide No. FZQ25465 (TJNHM).

**Diagnosis.** Adult (Figure 3E). Wingspan 10.0 mm. *Scrobipalpa picta* is distinguished by the forewing with a longitudinal dark brown band running from the base to the apex along the midline of the wing. The male genitalia are distinguished by the uncus subparallel sided in the basal half and narrowed from the middle to an almost straight apex, the inwardly curved valva inflated before the apex and reaching the apex of the uncus, and the junction between the sacculus and the vincular process with a small projection (Figure 5C).

**Distribution.** China (Xinjiang), Afghanistan [24].

**Note.** This species is newly recorded in China.

***Scrobipalpa subargenteonigra* Li, Bidzilya & Zhang sp. nov.**
(Figure 3F, Figure 5D and Figure 6E)
(LSIDurn:lsid:zoobank.org:act:BA5BD558-C173-44CB-BC75-62E781D38367)

**Type material.** Holotype ♂, **CHINA, Xinjiang:** Eastern Tamarix Forest, Halajun Village, Artux City, Kizilsu Kirgiz Autonomous Prefecture (40.18° N, 76.85° E), 1632.5 m, 25.v.2025, leg. XY Zhang et al., slide No. FZQ25383 (TJNHM).

Paratypes: **Xinjiang:** 4 ♂, same data as holotype, slide Nos. FZQ25407, ZYQ25255; 8♂13♀, Kan-Areli Village, Halajun Town, Artux City, Kizilsu Kirgiz Autonomous Prefecture (40.14° N, 76.78° E), 1626.2 m, 25.v.2025, leg. XY Zhang et al., slide Nos. FZQ25384♂, FZQ25405♂, FZQ25406♂, FZQ25400♀, FZQ25403♀, FZQ25404♀ (TJNHM); 1♂3♀, General Farm, Jiashi County (39.78° N, 77.55° E), 1157 m, 12.viii.2024, leg. GH Chen, QY Min and X Liu, slide No. ZYQ25132♀ (TJNHM); 4 ♀, Honghai Scenic Area, Bachu County (39.77° N, 78.29° E), 1127.1 m, 16.vi.2024, leg. XY Zhang, GH Chen and SL Zhang, slide No. ZYQ25136 (KSU).

**Diagnosis.** The new species is characterized externally by the black forewing having three creamy white fasciae, which is similar to *S. argenteonigra* Povolný, 1972 (Povolný 1972: 186, Figures 4, 18 and 34) [25]. It can be distinguished from *S. argenteonigra* in the male genitalia by the uncus parallel from near the base to before the obtuse apex, and the slender vincular process narrowed to a pointed apex; in the female genitalia it can be distinguished by the elongate colliculum that is 2/3 the length of the sternite VIII, and the signum situated at the entrance of the corpus bursae. In *S. argenteonigra*, the uncus is gradually narrowed from the base to the rounded apex, and the broader vincular process is narrowed to a hooked apex; the colliculum is much shorter, and the signum is situated at about the posterior 1/3 of the corpus bursae (Povolný 2002: 31, Figures 20, 291 and 766) [5].

**Description. Adult** (Figure 3F). Wingspan 10.0–11.0 mm.

*Head.* Vertex and frons creamy white. Antenna with scape dark brown; flagellum brown annulated with dark brown, gradually transitioning to creamy white annulated with dark brown. Labial palpus creamy white, except segment 2 brown at base on outer surface; segment 3 dark brown at apex.

*Thorax.* Mesonotum creamy white, tegula dark brown. Forewing black, with three creamy white fasciae; subbasal fascia narrow and short, extending to fold; antemedial fascia broad, slightly crossing fold; subterminal fascia longest, extending from costal margin to tornus; dorsum with a creamy white stripe at base, about same size as subbasal fascia; fringe greyish black. Hindwing greyish white; fringe grey.

*Abdomen.* Male genitalia (Figure 5D). Uncus subrectangular, parallel to before obtuse apex, 1.5 times as long as wide. Gnathos slender, slightly curved. Tegumen 2.5 times as long as wide, with a trapezoidal anteromedial emargination. Valva bent inward, inflated before apex, extending beyond apex of uncus. Sacculus 2/5 length of valva, of equal width, as wide as valva, with a denticle-like apical process directed inward. Vinculum with a broad U-shaped posteromedial emargination; vincular process slender, subtriangular, 1/2 length of sacculus, narrowed to pointed apex. Saccus broad at base, gradually narrowed to pointed apex, extending far beyond pedunculus. Phallus straight; caecum inflated, ca. 2/5 length of phallus; apical arm ca. 1/13 length of phallus.

Female genitalia (Figure 6E). Papilla analis subovate, with sparse short setae. Apophyses posteriores ca. four times longer than apophyses anteriores. Sternite VIII as long as midwidth, widened medially; subgenital plate with foamy sculptures from middle of inner margin to base of apophyses anteriores; ventromedial depression trapezoidal, divided by anteromedial triangular incision to 2/3 of its length, forming two very narrow lobes, each lobe with microspines and foamy sculptures, exceeding anterior margin of sternite VIII. Ductus bursae of equal width; colliculum long, 2/3 length of sternite VIII, anterior 1/3 sclerotized laterally. Corpus bursae elliptical; signum situated at entrance of corpus bursae, with distal hook weakly curved.

**Distribution.** China (Xinjiang).

**Etymology.** The specific epithet is derived from the Latin *sub* and the specific name of its similar species, indicating the similarities of the two species.

***Scrobipalpa xinjiangensis* Li, Bidzilya & Zhang sp. nov.**
(Figure 3G, Figure 5E and Figure 6F)
(LSIDurn:lsid:zoobank.org:act:0BE3123E-C496-41F1-B5A5-758390340124)

**Type material. CHINA, Xinjiang:** Holotype ♂, Kan-Areli Village, Halajun Town, Artux City, Kizilsu Kirgiz Autonomous Prefecture (40.14° N, 76.78° E), 1626.2 m, 25.v.2025, leg. ZQ Feng and C Cheng, slide No. FZQ25377 (TJNHM).

Paratypes: **Xinjiang:** 130♂96♀, same data as holotype, slide Nos. FZQ25375♂, FZQ25314♂, ZYQ25284♂, ZYQ25285♂, ZYQ25286♂, ZYQ25287♂, ZYQ25288♂, ZYQ25289♂, ZYQ25291♂, ZYQ25292♂, ZYQ25293♂, ZYQ25307♂, ZYQ25309♂, ZYQ25310♂, ZYQ25311♂, ZYQ25312♂, ZYQ25314♂, ZYQ25315♂, ZYQ25316♂, ZYQ25317♂, ZYQ25318♂, ZYQ25319♂, ZYQ25320♂, ZYQ25321♂, ZYQ25322♂, ZYQ25323♂, ZYQ25324♂, ZYQ25325♂, ZYQ25326♂, ZYQ25327♂, ZYQ25328♂, ZYQ25329♂, ZYQ25330♂, ZYQ25331♂, ZYQ25332♂, ZYQ25333♂, FZQ25376♂, FZQ25395♂, FZQ25229♀, FZQ25381♀, FZQ25382♀, ZYQ25308♀ (TJNHM and KSU); 7♂4♀, Eastern Tamarix Forest, Halajun Village, Artux City, Kizilsu Kirgiz Autonomous Prefecture (40.18° N, 76.85° E), 1632.5 m, 25.v.2025, leg. YQ Zhai and C Cheng, slide No. ZYQ25313♂ (TJNHM and KSU); 2 ♂, Honghai Scenic Area, Bachu County (39.77° N, 78.29° E), 1127.1 m, 16.vi.2024, leg. XY Zhang, GH Chen and SL Zhang, slide Nos. FZQ25222, FZQ24187 (TJNHM).

**Diagnosis.** The new species resembles *S. occulta* Povolný, 2002 and *S. sibirica* Bidzilya, 2009 externally and in the genitalia. It can be separated from the latter two species by the presence of a distinct pale subapical fascia on the forewing, the narrow and long sacculus that exceeds the apex of the vincular process in male genitalia, and the ventromedial depression covered with foam sculptures in the female genitalia. In *S. occulta* and *S. sibirica*, the forewings have an indistinct subapical fascia, the wider and shorter sacculus does not exceed the apex of the vincular process in the male genitalia, and the ventromedial depression is covered with microspines in the female genitalia (Huemer and Karsholt, 2010: 80, Figure 27; Bidzilya, 2009: 9, Figures 6, 14 and 19) [13,22].

The differences between this new species and *S. latiuscula* sp. nov. are stated in the diagnosis of the preceding species.

**Description. Adult** (Figure 3G). Wingspan 13.0–13.5 mm.

*Head.* Vertex and frons cream, mixed with pale brown scales. Antenna with scape dark brown, cream at apex; flagellum light brown with dark brown basal rings. Labial palpus off-white, segment 2 with scattered, grey-tipped scales on ventral surface; segment 3 ringed with black.

*Thorax.* Mesonotum and tegula cream, mixed with brown scales. Forewing cream, evenly covered with dense brown spots and seven black spots: costal margin with black spot near base and at basal 1/4, fold with black spot at base, near base and middle, cell with black spot at middle and end, respectively; cream subterminal fascia from basal 3/4 of costal margin zigzag to dorsum; fringe yellowish grey. Hindwing white, fringe light yellowish grey.

*Abdomen.* Male genitalia (Figure 5E). Uncus subtrapezoidal, slightly longer than proximal width, rounded apex. Gnathos short, slightly curved. Tegumen twice as long as midwidth, with a broad shallow semicircular anteromedial emargination. Valva narrow, of equal width, curved inward, apex pointed, extending beyond apex of uncus. Sacculus ca. 1/3 length of valva, apex pointed and curved inward. Vinculum with a broad V-shaped posterior emargination; vincular process broadly inflated, with dense setae. Saccus wide at base, narrowed to rounded apex, extending far beyond pedunculus apically. Phallus straight; caecum inflated, ca. 1/2 length of phallus; apical arm thick, leaf-shaped, curved upward, ca. 1/6 length of phallus.

Female genitalia (Figure 6F). Papilla analis subovate, with sparse short setae. Apophyses posteriores five times longer than apophyses anteriores. Sternite VIII trapezoidal; subgenital plate 1/4 width of sternite VIII, covered with fine microspines from outer margin to half width of subgenital plate, bearing foamy sculptures along inner margin from posterior 2/5 to base of apophyses anteriores, extending slightly beyond anterior margin of sternite VIII; ventromedial depression trapezoidal, divided by triangular anteromedial incision to 1/2 length, forming two subovate lobes, each lobe with foamy sculptures, as wide as subgenital plate. Ductus bursae narrow at base, distally broadened towards elliptical corpus bursae; colliculum near base of ductus bursae. Corpus bursae elliptical; signum situated near entrance of corpus bursae, ca. 1/4 length of corpus bursae, distal hook weakly curved.

**Distribution.** China (Xinjiang).

**Etymology.** The specific epithet is from the type locality, Xinjiang Uygur Autonomous Region, China.

***Scrobipalpa zaitzevi* Piskunov, 1990**
(Figure 3H and Figure 5F)

*Scrobipalpa zaitzevi* Piskunov, 1990: 306. TL: Mongolia. TD: ZIRAS (= ZIN) [26].

*Ilseopsis punctata* Povolný, 1996: 26 [27].

*Euscrobipalpa zaitzevi*: Povolný, 2002: 78 [5].

*Scrobipalpa zaitzevi*: Bidzilya, Huemer & Šumpich, 2022: 16 [6].

**Material examined. CHINA, Xinjiang:** 1 ♂, Postan Terek Township, Wuqia County, Kizilsu Kirgiz Autonomous Prefecture (39.26° N, 75.07° E), 2141 m, 25.vi.2025, leg. XY Zhang and XH Liu, slide No. FZQ25679 (TJNHM).

**Diagnosis.** Adult (Figure 3H). Wingspan 10.0 mm. *Scrobipalpa zaitzevi* is distinguished by the creamy white forewing evenly covered with dense brown scales and with ill-defined black spots. The male genitalia are distinguished by the extremely broad uncus gradually narrowed to before the apex and with prominently folded plates laterodistally, the uniformly narrow valva, the triangular sacculus, and the outwardly hooked vincular process (Figure 5F).

**Distribution.** China (Xinjiang), Russia (Altai, S Ural) (Bidzilya et al. 2022: 17; Junnilainen et al. 2010: 50) [6,28]; Kyrgyzstan (Povolný 1996: 26) [27]; Tajikistan, Afghanistan (Bidzilya et al. 2022: 17) [6]; Mongolia (Piskunov 1990: 306) [27].

**Note.** This species is newly recorded in China.

**Checklist of the genus *Scrobipalpa* in Xinjiang, China**
**1. *Scrobipalpa ahasver* Povolný, 1969**
  *Scrobipalpa ahasver* Povolný, 1969a: 5. TL: Mongolia. TD: HNHM [15].
  *Euscrobipalpa ahasver*: Povolný, 2002: 29 [5].
  *Scrobipalpa ahasver*: Li & Bidzilya 2019: 127 [11].
  **Distribution.** China (Xinjiang), Mongolia (Li and Bidzilya 2019) [11].
**2. *Scrobipalpa* *apicidentata* Li, Bidzilya & Zhang sp. nov.**
  **Distribution.** China (Xinjiang).
**3. *Scrobipalpa aptatella* (Walker, 1864)**
  *Gelechia aptatella* Walker, 1864: 636. TL: Sri Lanka. TD: NHMUK [29].
  *Gelechia heliopa* Lower, 1900: 417 [30].
  *Scrobipalpa heliopa*: Povolný, 1964: 350 [31].
  *Scrobipalpa aptatella*: Bidzilya & Li, 2010: 2 [10].
  **Host plants.** Solanaceae: *Datura innoxia* Mill., *Nicotiana tabacum* L., *N. rustica* L., *Solanum dubium* Fresen., *S. melongena* L., *S. tuberosum* L. (Huemer and Karsholt 2010) [13].
  **Distribution.** China (Xinjiang), E. Australia, introduced into the Oriental and Afrotropical regions. Records from Europe are unconfirmed (Bidzilya and Li 2010) [10].
**4. *Scrobipalpa artemisiella* (Treitschke, 1833)**
  *Lita artemisiella* Treitschke, 1833: 97. TL: Germany. TD: HNHM [32].
  *Anacampsis ancillella* Bruand, 1851: 41 [33].
  *Scrobipalpa gregori* Povolný, 1967: 218 [4].
  *Scrobipalpa artemisiella syriaca* Povolný, 1967: 214 [4].
  *Scrobipalpa artemisiella mongolensis* Povolný, 1969a: 6 [15].
  *Ilseopsis artemisiella*: Povolný, 1998: 339 [34].
  *Euscrobipalpa artemisiella*: Powell & Povolný, 2001: 19 [35].
  *Euscrobipalpa artemisiella mongolensis* Povolný, 2002: 32 [5].
  *Scrobipalpa artemisiella*: Lee et al., 2009: 26 [9].
  **Distribution.** Palaearctic region eastwards to China (Ningxia, Xinjiang) and Mongolia (Li and Bidzilya 2019) [11].
**5. *Scrobipalpa atriplicella*** **(Fischer von Röslerstamm, 1841)**
  *Lita atriplicella* Fischer von Röslerstamm, 1841: 223. TL: Norway. TD: NHMW [36].
  *Gnorimoschema chenopodiella* Busck, 1916: 148 [37].
  *Scrobipalpa (Euscrobipalpa) arogantella* Povolný, 1967: 213 [4].
  *Scrobipalpa altajica* Povolný, 1969a: 5 [15].
  *Ilseopsis atriplicella*: Povolný, 1998: 339 [34].
  *Euscrobipalpa atriplicella*: Powell & Povolný, 2001: 20 [35].
  *Scrobipalpa atriplicella*: Huemer & Karsholt, 2010: 128 [13].
  **Host plants.** Amaranthaceae: *Atriplex laciniata* L., *A. patula* L., *A. prostrata* DC, *A. tatarica* L., *Beta vulgaris* L., *Chenopodium album* L., *C. ficifolium* Sm., *C. hybridum* L., *C. murale* L., *C. quinoa* Willd., *Halimione portulacoides* L. (Huemer and Karsholt 2010) [13].
  **Distribution.** Palaearctic region eastwards to eastern China (Henan, Heilongjiang, Inner Mongolia, Jilin, Qinghai, Shaanxi, Sichuan, Tibet, Xinjiang), USA (introduced) (Bidzilya and Li 2010; Huemer and Karsholt 2010) [10,13].
**6. *Scrobipalpa autonoma* Povolný, 1969**
  *Scrobipalpa autonoma* Povolný, 1969a: 20. TL: Mongolia. TD: HNHM [15].
  *Euscrobipalpa autonoma*: Povolný, 2002: 33 [5].
  **Distribution.** China (Xinjiang), Mongolia (Povolný 2002) [5].
  **Note.** This species is newly recorded in China.
**7. *Scrobipalpa bryophiloides* Povolný, 1966**
  *Scrobipalpa bryophiloides* Povolný, 1966a: 397. TL: Kazakhstan. TD: MfN (= ZMB) [21].
  *Euscrobipalpa bryophiloides*: Povolný, 2002: 36 [5].
  *Scrobipalpa bryophiloides*: Bidzilya & Li, 2010: 2 [10].
  **Host plant.** Amaranthaceae: *Suaeda confusa* Iljin (Falkovitsh and Bidzilya 2006) [16].
  **Distribution.** China (Inner Mongolia, Ningxia, Shaanxi, Xinjiang), Eastern and Northern Europe, Turkey, Uzbekistan, Iran, Mongolia, SE Kazakhstan, Turkmenistan (Huemer and Karsholt 2010; Li and Bidzilya 2019) [11,13].
**8. *Scrobipalpa candicans* (Povolný, 1996)**
  *Ilseopsis candicans* Povolný, 1996: 20. TL: Kyrgyzstan. TD: UZMH [27].
  *Euscrobipalpa candicans*: Povolný, 2002: 37 [5].
  *Scrobipalpa candicans*: Bidzilya & Li, 2010: 2 [10].
  **Distribution.** China (Xinjiang), Kyrgyzstan, SE Kazakhstan (Li and Bidzilya 2019) [11].
**9.** ***Scrobipalpa concerna* Povolný, 1969**
  *Scrobipalpa concerna* Povolný, 1969a: 21. TL: Mongolia. TD: HNHM [15].
  *Euscrobipalpa concerna*: Povolný, 2002: 37 [5].
  *Scrobipalpa concerna uzbeka* Falkovitsh & Bidzilya, 2006: 70 [16].
  **Distribution.** China (Xinjiang), Mongolia, Uzbekistan [5,16].
  **Note.** This species is newly recorded in China.
**10. *Scrobipalpa dalibori* Lvovsky & Piskunov, 1989**
  *Scrobipalpa dalibori* Lvovsky & Piskunov, 1989: 537. TL: Mongolia. TD: ZIRAS (= ZIN) [17].
  *Euscrobipalpa cultrata* Lvovsky & Piskunov, 1989, Povolný, 2002: 39 [5].
  **Distribution.** China (Xinjiang), Mongolia [17].
  **Note.** This species is newly recorded in China.
**11. *Scrobipalpa divergens* (Povolný, 2002)**
  *Euscrobipalpa divergens* Povolný, 2002: 40. TL: China (Xinjiang). TD: ZMHU [5].
  *Scrobipalpa divergens*: Bidzilya & Li, 2010: 4 [10].
  **Distribution.** China (Xinjiang) (Povolný 2002) [5].
**12. *Scrobipalpa erichi* Povolný, 1964**
  *Scrobipalpa erichi* Povolný, 1964: 356. TL: Moldova. Coll: Hering [31].
  *Euscrobipalpa erichi*: Povolný, 2002: 41 [5].
  *Scrobipalpa erichi*: Bidzilya & Li, 2010: 4 [10].
  **Host plants.** Solanaceae: *Lycium barbarum* L., *Nicotiana tabacum* L. (Huemer and Karsholt 2010) [13].
  **Distribution.** China (Inner Mongolia, Xinjiang), Central (Austria) and Eastern Europe, Iran, Turkey, Middle Asia, Mongolia (Huemer and Karsholt 2010; Li and Bidzilya 2019) [11,13].
**13. *Scrobipalpa erichiodes* Bidzilya & Li, 2010**
  *Scrobipalpa erichiodes* Bidzilya & Li, 2010: 14. TL: China. TD: NKU [10].
  **Host plant.** Solanaceae: *Lycium barbarum* L. (Bidzilya and Li 2010) [10].
  **Distribution.** China (Gansu, Hebei, Heilongjiang, Inner Mongolia, Ningxia, Shaanxi, Xinjiang) (Li and Bidzilya 2019) [11].
**14. *Scrobipalpa frugifera* Povolný, 1969**
  *Scrobipalpa frugifera* Povolný, 1969a: 8. TL: Mongolia. TD: HNHM [15].
  *Scrobipalpa hypothetica* Povolný, 1973a: 20 [38].
  *Euscrobipalpa frugifera*: Povolný, 2002: 42 [5].
  *Scrobipalpa frugifera*: Li & Bidzilya 2019: 122 [11].
  **Distribution.** China (Inner Mongolia, Xinjiang) (Li and Bidzilya 2019) [11], Russia (Kalmykia, Altai, Tuva, South of Krasnoyarskiy krai, Buryatia, Zabaikalskiy krai, Khabarovskiy krai) (Ponomarenko 2008: 96; Bidzilya 2009: 5) [22,39], Kazakhstan (Bidzilya, Huemer and Šumpich 2022) [6], Kyrgyzstan (Povolný 1996) [27], Mongolia (Povolný 1969b) [24].
**15. *Scrobipalpa fusca* Bidzilya & Li, 2010**
  *Scrobipalpa fusca* Bidzilya & Li, 2010: 17. TL: China. TD: NKU [10].
  **Distribution.** China (Inner Mongolia, Xinjiang), Kazakhstan, Turkmenistan, Uzbekistan (Li and Bidzilya 2019) [11].
**16. *Scrobipalpa geomicta* (Meyrick, 1918)**
  *Phthorimaea geomicta* Meyrick, 1918: 18. TL: South Africa. TD: TMSA [19].
  *Phthorimaea vicaria* Meyrick, 1921: 74 [20].
  *Scrobipalpa geomicta*: Janse, 1951: 208 [14].
  *Scrobipalpa vicaria* (Meyrick, 1921): Janse, 1951: 220 [14].
  *Scrobipalpa tineiformis* Povolný, 1967: 230 [4].
  *Scrobipalpa geomict* Bidzilya 2021: 36 [1].
  **Distribution.** China (Xinjiang), S. Europe (Spain, Greece, Malta, Cyprus), N. Africa, Mongolia, W. India, Ethiopia, S. Africa, and Namibia (Bidzilya 2021) [1]; Libya, Cyprus, Saudi Arabia, Iran, Pakistan (Huemer and Karsholt 2010) [13].
  **Note.** This species is newly recorded in China.
**17. *Scrobipalpa gobica* Povolný, 1969**
  *Scrobipalpa gobica* Povolný, 1969a: 9. TL: Mongolia. TD: HNHM [15].
  *Euscrobipalpa gobica*: Povolný, 2002: 44 [5].
  *Scrobipalpa gobica*: Bidzilya & Li, 2010: 4 [10].
  **Distribution.** China (Xinjiang) (Li and Bidzilya 2019) [11], Mongolia (Povolný 1969a) [15].
**18. *Scrobipalpa gozmanyi* Povolný, 1969**
  *Scrobipalpa gozmanyi* Povolný, 1969a: 10. TL: Mongolia. TD: HNHM [15].
  *Euscrobipalpa gozmanyi*: Povolný, 2002: 44 [5].
  *Scrobipalpa gozmanyi*: Bidzilya & Li, 2010: 4 [10].
  **Distribution.** China (Gansu, Ningxia, Qinghai, Sichuan, Xinjiang) (Li and Bidzilya 2019) [11], Mongolia (Povolný 1969a) [15].
**19. *Scrobipalpa grisea* Povolný, 1969**
  *Scrobipalpa grisea* Povolný, 1969a: 10. TL: Mongolia. TD: HNHM [15].
  *Euscrobipalpa grisea*: Povolný, 2002: 44 [5].
  *Scrobipalpa grisea*: Bidzilya & Li, 2010: 4 [10].
  **Distribution.** China (Inner Mongolia, Xinjiang) (Li and Bidzilya 2019) [11], Mongolia (Povolný 1969a) [15], Russia (South Ural, South Siberia) (Povolný 2002; Ponomarenko 2019) [5,23], S. Korea (Park and Ponomarenko 2006) [40].
**20. *Scrobipalpa halimioniella* Huemer & Karsholt, 2010**
  *Scrobipalpa halimioniella* Huemer & Karsholt, 2010: 182. TL: France. TD: ZMUC and TLMF [13].
  **Host plant.** Amaranthaceae: *Halimione portulacoides* (L.) (Bidzilya and Li 2010) [10].
  **Distribution.** China (Xinjiang) (Li and Bidzilya 2019) [11], S. France, S. Ukraine (Bidzilya et al. 2011) [41].
**21. *Scrobipalpa hannemanni* Povolný, 1966**
  *Scrobipalpa hannemanni* Povolný, 1966a: 402. TL: Croatia. TD: MfN (= ZMB) [21].
  *Scrobipalpa hannemanni furva* Povolný, 1969a: 12 [15].
  *Euscrobipalpa hannemanni hannemanni* (Povolný, 1966), Povolný, 2002: 46 [5].
  *Euscrobipalpa hannemanni gamanthi* Falkovitsh & Bidzilya, 2006: 77 [16].
  *Scrobipalpa hannemanni*: Huemer & Karsholt, 2010: 168 [13].
  **Distribution.** China (Xinjiang), Croatia (Povolný 1966a) [21], Russia (Lower Volga region, S Ural, Zabaikalskiy krai) (Bidzilya 2009; Ponomarenko 2019) [22,23]. Subspecies *S. h. furva* Povolný, 1969 in Mongolia [5], and subspecies *S. h. gamanthi* Falkovitsh & Bidzilya, 2006 in Uzbekistan [16].
  **Note.** This species is newly recorded in China.
**22. *Scrobipalpa heretica* Povolný, 1973**
  *Scrobipalpa heretica* Povolný, 1973b: 107. TL: Turkey. Coll: W. Glaser (GI) [42].
  *Scrobipalpa submagnificella* Povolný, 1977: 330 [43].
  *Euscrobipalpa heretica*: Povolný, 2002: 46 [5].
  *Scrobipalpa heretica*: Li & Bidzilya 2019: 127 [11].
  **Distribution.** China (Xinjiang) (Li and Bidzilya 2019) [11], Spain (Huemer and Karsholt 2010: 109) [13], Russia (Volga and Ural regions, Altai, Buryatia) (Ponomarenko 2019; Bidzilya, Huemer and Šumpich, 2022) [6,23], Afghanistan, Kazakhstan, Kyrgyzstan (Povolný 2002) [5].
**23. *Scrobipalpa indignella* (Staudinger, 1879)**
  *Bryotropha indignella* Staudinger, 1879: 308. TL: Turkey. TD: ZMHU [44].
  *Gnorimoschema pseudobsoletellum* Povolný & Gregor, 1955: 83 [45].
  *Scrobipalpa grossa* Povolný, 1966a: 400 [21].
  *Euscrobipalpa grossa*: Povolný, 1969b: 379 [24].
  *Scrobipalpa pseudobsoletella* Povolný, 1976: 46 [46].
  *Euscrobipalpa indignella*: Povolný, 2002: 48 [5].
  *Scrobipalpa indignella*: Karsholt & Rutten, 2005: 80; Bidzilya & Li, 2010: 5 [10].
  **Distribution.** China (Xinjiang), Turkey, Afghanistan, Turkmenistan, Syria, Azerbaijan, Russia (SE of the European part), S. Ukraine (Li and Bidzilya 2019) [11].
**24. *Scrobipalpa intricata* Povolný, 1969**
  *Scrobipalpa intricata* Povolný, 1969a: 11. TL: Mongolia. TD: HNHM [15].
  *Euscrobipalpa intricata*: Povolný, 2002: 50 [5].
  *Scrobipalpa intricata*: Li & Bidzilya 2019: 120 [11].
  **Distribution.** China (Xinjiang) (Li and Bidzilya 2019) [11], Mongolia (Povolný 1969a) [15].
**25. *Scrobipalpa kaszabi* Povolný, 1969**
  *Scrobipalpa kaszabi* Povolný, 1969a: 12. TL: Japan. TD: HNHM [15].
  *Euscrobipalpa kurokoi*: Povolný, 2002: 53 [5].
  *Scrobipalpa kaszabi*: Li & Bidzilya 2019: 122 [11].
  **Distribution.** China (Inner Mongolia, Gansu, Xinjiang) (Li and Bidzilya 2019) [11], Mongolia (Povolný 1969a) [15].
**26. *Scrobipalpa latiuscula* Li, Bidzilya & Zhang sp. nov.**
  **Distribution.** China (Xinjiang).
**27. *Scrobipalpa magnificella* Povolný, 1967**
  *Scrobipalpa magnificella* Povolný, 1967: 221. TL: Syria. TD: NHM [4].
  *Euscrobipalpa magnificella*: Povolný, 2002: 54 [5].
  *Scrobipalpa magnificella*: Bidzilya & Li, 2010: 5 [10].
  **Distribution.** China (Xinjiang), S. Ukraine, Russia (southern Ural), Syria, north Iran, Uzbekistan, Mongolia (Li and Bidzilya 2019) [11].
**28. *Scrobipalpa maniaca* Povolný, 1969**
  *Scrobipalpa maniaca* Povolný, 1969a: 13. TL: Mongolia. TD: HNHM [15].
  *Scrobipalpa turkmeniella* Piskunov, 1973: 1579 [47]. [In Russian]
  *Euscrobipalpa maniaca*: Povolný, 2002: 54 [5].
  *Scrobipalpa maniaca*: Bidzilya & Li, 2010: 5 [10].
  **Distribution.** China (Xinjiang), Afghanistan, Mongolia, Russia (Lower Volga region, Zabaikalskiy krai), Turkmenistan, Uzbekistan (Li and Bidzilya 2019) [11].
**29. *Scrobipalpa mongolica* Povolný, 1969**
  *Scrobipalpa mongolica* Povolný, 1969a: 14. TL: Mongolia. TD: HNHM [15].
  *Euscrobipalpa mongolica*: Povolný, 2002: 56 [5].
  *Scrobipalpa mongolica*: Bidzilya & Li, 2010: 5 [10].
  **Distribution.** China (Inner Mongolia, Qinghai, Xinjiang), Mongolia (Li and Bidzilya 2019) [11].
**30. *Scrobipalpa mongoloides* Povolný, 1969**
  *Scrobipalpa mongoloides* Povolný, 1969a: 15. TL: Mongolia. TD: HNHM [15].
  *Euscrobipalpa mongoloide*: Povolný, 2002: 56 [5].
  *Scrobipalpa mongoloides*: Bidzilya & Li, 2010: 5 [10].
  **Distribution.** China (Gansu, Hebei, Henan, Inner Mongolia, Ningxia, Qinghai, Xinjiang), Kazakhstan, Kyrgyzstan, N. Pakistan, Uzbekistan, Mongolia (Li and Bidzilya 2019) [11].
**31. *Scrobipalpa nigrosparsea* Povolný, 1969**
  *Scrobipalpa nigrosparsea* Povolný, 1969a: 16. TL: Mongolia. TD: HNHM [15].
  *Euscrobipalpa nigrosparsea*: Povolný, 2002: 57 [5].
  *Scrobipalpa nigrosparsea*: Bidzilya & Li, 2010: 6 [10].
  **Distribution.** China (Inner Mongolia, Xinjiang) (Li and Bidzilya 2019) [11], Mongolia (Povolný 1969a) [15].
**32. *Scrobipalpa nitentella* (Fuchs, 1902)**
  *Lita nitentella* Fuchs, 1902: 324. TL: Germany. TD: DEI [48].
  *Phthorimaea seminella* Pierce & Metcalfe, 1935: 98 [49].
  *Phthorimaea seminella* f. *spinosa* Gaede, 1937: 286 [50].
  *Lita nitentella*: Sattler, 1961: 30 [51].
  *Scrobipalpa nitentella*: Povolný, 1966b: 139 [52].
  *Euscrobipalpa nitentella*: Povolný, 2002: 58 [5].
  *Scrobipalpa nitentella*: Bidzilya & Li, 2010: 6 [10].
  **Host plants.** Amaranthaceae: *Atriplex hastata* L., *A*. *hortensis* L., *A*. *littoralis* L., *A*. *praecox* Hülph., *A*. *prostrata* DC., *Beta maritima* (L.), *Chenopodium album* L., *Halimione pedunculata* (L.), *H*. *portulacoides* (L.), *Salicornia europaea* L., *Suaeda altissima* (L.), *S*. *maritima* (L.) (Huemer and Karsholt 2010) [13].
  **Distribution.** China (Qinghai, Xinjiang), Europe, Turkey, Kazakhstan, Afghanistan, Russia (European part, Novosibirsk region, Zabaikalskiy krai), Mongolia (Li and Bidzilya 2019) [11].
**33. *Scrobipalpa obsoletella* (Fischer von Röslerstamm, 1841)**
  *Lita obsoletella* Fischer von Röslerstamm, 1841: 225. TL: England. TD: NHMUK [36].
  *Gnorimoschema miscitatella* Clarke, 1932: 66 [53].
  *Phthorimaea bipunctella* Hartig, 1941: 158 [54].
  *Phthorimaea calaritanella* Amsel, 1952: 128 [55].
  *Scrobipalpa obsoletella hospes* Povolný, 1964: 354 [31].
  *Euscrobipalpa obsoletella*: Powell & Povolný, 2001: 21 [35].
  *Scrobipalpa obsoletella*: Huemer & Karsholt, 2010: 86 [13].
  **Host plants.** Amaranthaceae: *Atriplex glabriuscula* Edmondston, *A. halimus* L., *A. littoralis* L., *A. tatarica* L. (Huemer and Karsholt 2010) [13].
  **Distribution.** Palaearctic region eastwards to China (Gansu, Inner Mongolia, Xinjiang) and Mongolia, S. Africa, USA (introduced) (Huemer and Karsholt 2010) [13].
**34. *Scrobipalpa occulta* (Povolný, 2002)**
  *Euscrobipalpa occulta* Povolný, 2002: 59. TL: Turkey. TD: SMNK [5].
  *Scrobipalpa sibirica* Bidzilya, 2009: 9 [22].
  *Scrobipalpa occulta*: Huemer & Karsholt, 2010: 80 [13].
  **Distribution.** China (Xinjiang) (Li and Bidzilya 2019) [11], Russia (S. Ural) (Huemer and Karsholt 2010) [13], Kazakhstan (Bidzilya et al. 2022) [6].
**35. *Scrobipalpa parki* Povolný, 1993**
  *Ilseopsis parki* Povolný, 1993: 355. TL: South Korea. TD: CIS [56].
  *Euscrobipalpa parki*: Povolný, 2002: 61 [5].
  *Scrobipalpa parki*: Bidzilya & Li, 2010: 6 [10].
  **Host plants.** Solanaceae: *Lycium barbarum* L., *L*. *chinense* Mill. (Bidzilya and Li 2010) [10].
  **Distribution.** China (Gansu, Hebei, Ningxia, Qinghai, Shaanxi, Xinjiang), S. Korea (Li and Bidzilya 2019 [11].
**36. *Scrobipalpa perfecta* (Povolný, 1996)**
  *Ilseopsis perfecta* Povolný, 1996: 24. TL: Kazakhstan. TD: UZMH [27].
  *Euscrobipalpa perfecta*: Povolný, 2002: 61 [5].
  *Scrobipalpa perfecta*: Li & Bidzilya 2019: 121 [11].
  **Distribution.** China (Xinjiang) (Li and Bidzilya 2019) [11], Kyrgyzstan (Povolný 1996) [27].
**37. *Scrobipalpa picta* Povolný, 1969**
  *Scrobipalpa (Euscrobipalpa) picta* Povolný, 1969b: 375. TL: Afghanistan. TD: SMNK (=LNK) [24].
  *Euscrobipalpa picta*: Povolný, 2002: 62 [5].
  **Distribution.** China (Xinjiang), Afghanistan (Povolný 1969b) [24].
  **Note.** This species is newly recorded in China.
**38. *Scrobipalpa pulchra* Povolný, 1967**
  *Scrobipalpa pulchra* Povolný, 1967: 223. TL: Afghanistan. TD: NHMW [4].
  *Euscrobipalpa pulchra*: Povolný, 2002: 64 [5].
  *Scrobipalpa pulchra*: Bidzilya & Li, 2010: 6 [10].
  **Host plants.** Amaranthaceae: *Climacoptera crassa* (Bieb.), *Haloxylon ammodendron* (C. A. Mey.), *H*. *persicum* Bunge ex Boiss. & Buhse; Amaryllidaceae: *Gamanthus gamocarpus* (Moq.) (Huemer and Karsholt 2010) [13].
  **Distribution.** China (Xinjiang) (Li and Bidzilya 2019) [11], Latvia, Mongolia, S. Ukraine, Turkey, Israel (Huemer and Karsholt 2010) [13].
**39. *Scrobipalpa sattleri* Lvovsky & Piskunov, 1989**
  *Scrobipalpa sattleri* Lvovsky & Piskunov, 1989: 542. TL: Mongolia. TD: ZIRAS (=ZIN) [17].
  *Euscrobipalpa sattleri*: Povolný, 2002: 68 [5].
  *Scrobipalpa sattleri*: Bidzilya & Li, 2010: 7 [10].
  **Distribution.** China (Inner Mongolia, Xinjiang) (Bidzilya and Li 2010) [10], Mongolia (Lvovsky and Piskunov 1989) [17].
**40. *Scrobipalpa selectella* (Caradja, 1920)**
  *Gelechia selectella* Caradja, 1920: 99. TL: Kazakhstan. TD: MINGA [57].
  *Scrobipalpa fraterna* Povolný, 1969a: 8 [15].
  *Scrobipalpa selectella*: Huemer & Karsholt, 2010: 118 [13].
  **Host plants.** Amarantaceae: *Halostachys belangeriana* (Moq.) Botsch., *Halocnemum strobilaceum* (Pall.) Bieb. (Huemer and Karsholt 2010) [13].
  **Distribution.** China (Xinjiang, Ningxia, Inner Mongolia, Tianjin) (Li and Bidzilya 2019) [11], Mongolia, Turkey, South of European Russia, Ukraine, Greece, Tunisia (Huemer and Karsholt 2010) [13].
**41. *Scrobipalpa similis* Povolný, 1973**
  *Scrobipalpa similis* Povolný, 1973b: 21. TL: Mongolia. TD: SMNK [41].
  *Euscrobipalpa kyrana* Povolný, 2001: 193 [58].
  *Euscrobipalpa similis*: Povolný, 2002: 79 [5].
  *Scrobipalpa similis*: Bidzilya & Li, 2010: 7 [10].
  **Distribution.** China (Gansu, Xinjiang), Mongolia, Russia (Zabaikalskiy krai), SE Kazakhstan (Li and Bidzilya 2019) [11].
**42. *Scrobipalpa solitaria* Povolný, 1969**
  *Scrobipalpa solitaria* Povolný, 1969a: 21. TL: Mongolia. TD: HNHM [15].
  *Euscrobipalpa solitaria*: Povolný, 2002: 70 [5].
  *Scrobipalpa solitaria*: Bidzilya & Li, 2010: 7 [10].
  **Distribution.** China (Xinjiang), Ukraine, Russia (S. Ural), Mongolia (Li and Bidzilya 2019) [11].
**43. *Scrobipalpa suaveolens* (Povolný, 1996)**
  *Ilseopsis* (*Euscrobipalpa*) *suaveolens* Povolný, 1996: 27. TL: Kyrgyzstan. TD: UZMH [27].
  *Euscrobipalpa suaveolens*: Povolný, 2002: 72 [5].
  *Scrobipalpa suaveolens*: Li & Bidzilya 2019: 121 [11].
  **Distribution.** China (Xinjiang) (Li and Bidzilya 2019) [11], Kyrgyzstan (Povolný 1996) [27].
**44. *Scrobipalpa subargenteonigra* Li, Bidzilya & Zhang sp. nov.**
  **Distribution.** China (Xinjiang).
**45. *Scrobipalpa subnitens* Povolný, 1969**
  *Scrobipalpa subnitens* Povolný, 1969a: 18. TL: Mongolia. TD: SMNK (= LNK) [15].
  *Scrobipalpa gorodkovi* Bidzilya, 2012: 158 [59].
  *Scrobipalpa subnitens*: Li & Bidzilya 2019: 123 [11].
  **Distribution.** China (Ningxia, Xinjiang), Tajikistan (Li and Bidzilya 2019) [11], Mongolia (Povolný 1969a) [15].
**46. *Scrobipalpa vartianorum* Povolný, 1968**
  *Scrobipalpa vartianorum* Povolný, 1968: 16. TL: Iran [18]. TD: ?
  *Euscrobipalpa vartianorum*: Povolný, 2002: 76 [5].
  *Scrobipalpa vartianorum*: Bidzilya & Li, 2010: 8 [10].
  **Distribution.** China (Xinjiang), Iran (Bidzilya and Li 2010) [10].
**47. *Scrobipalpa xinjiangensis* Li, Bidzilya & Zhang sp. nov.**
  **Distribution.** China (Xinjiang).
**48. *Scrobipalpa zaitzevi* Piskunov, 1990**
  *Scrobipalpa zaitzevi* Piskunov, 1990: 306. TL: Mongolia. TD: ZIRAS (=ZIN) [26].
  *Euscrobipalpa zaitzevi*: Povolný, 2002: 78 [5].
  *Ilseopsis punctata* Povolný, 1996: 26 [27].
  *Scrobipalpa zaitzevi*: Bidzilya, Huemer & Šumpich, 2022: 16 [6].
  **Distribution.** China (Xinjiang), Russia, Altai, Kyrgyzstan, Tajikistan, Afghanistan, Mongolia (Bidzilya et al. 2022) [6].
  **Note.** This species is newly recorded in China.

## 4. Discussion

Based on external morphological characteristics, this study presents a taxonomic study of the genus *Scrobipalpa* from Xinjiang, China. Specimens collected from various localities across Xinjiang were examined using morphological observation and genitalia dissection. A total of four new species and seven newly recorded species for China are identified, and a list of 48 *Scrobipalpa* species known from Xinjiang is provided.

The genus *Scrobipalpa* is a species-rich group within the tribe Gnorimoschemini of the family Gelechiidae, showing a distribution pattern of enrichment in the arid regions. In the Palaearctic Region, *Scrobipalpa* are most diverse in the open arid landscapes. They are the dominant gelechiids in halophytic habitats, dry mountains steppes, deserts, and semideserts [52].

Currently, a total of 87 species of the genus Scrobipalpa are known in China. Xinjiang accounts for about one-sixth of China’s land area, mostly characterized by arid and semi-arid environments. From the perspective of zoogeography, Xinjiang provides diverse habitats for *Scrobipalpa*, promoting population isolation and speciation.

Among the new species, *Scrobipalpa latiuscula* sp. nov., *S. subargenteonigra* sp. nov., and *S. xinjiangensis* sp. nov. are concentrated in the western section of the southern Tianshan Mountains (Artux City, Wuqia County) and the northwestern margin of the Tarim Basin. The remaining new species, *S. apicidentata* sp. nov., is currently known only from the southwestern margin of the Tarim Basin (Pishan County, Yecheng County). This region is a typical representative of the Central Asian arid zone, characterized by abundant desert shrubland and Gobi habitats.

Among the newly recorded species, *Scrobipalpa autonoma* Povolný, 1969, *S. concerna* Povolný, 1969, *S. dalibori* Lvovsky & Piskunov, 1989, and *S. zaitzevi* Piskunov, 1990 are all known to occur in Mongolia [5,17,27]; *S. hannemanni* Povolný, 1966 is distributed in Croatia, Russia, Mongolia, and Uzbekistan [5,16,21,22]; *S. picta* Povolný, 1969 is distributed in Afghanistan [24]; and *S. geomicta* (Meyrick, 1918) is widely distributed in Southern Europe, North Africa, the Middle East, South Asia, and southern Africa [1]. The discovery of these species in Xinjiang further confirms the region as a key area for species dispersal and convergence in the arid eastern Palaearctic Region.

The specimens collected in this study range in altitude from 263 m (Jinghe County) to 3727 m (Taxkorgan County), demonstrating the strong altitudinal adaptability of the genus, although vertical distribution patterns differ significantly among species.

Low-altitude desert species (alt. < 500 m): *Scrobipalpa geomicta* (Meyrick, 1918) (alt. 877–1736 m), *S. xinjiangensis* sp. nov. (alt. 1127–1632 m), and *S. subargenteonigra* sp. nov. (alt. 1127–1632 m). The habitats in this region are mostly desert shrublands (e.g., tamarisk forests, saline deserts).

Mid-altitude montane species (alt. 1500–2500 m): *S. apicidentata* sp. nov. (alt. 1601–2881 m) and *S. autonoma* Povolný, 1969 (alt. 1626–3727 m). The habitats in this region are mostly montane grasslands, gravel Gobi, and river valley shrublands.

High-altitude species (alt. > 2500 m): *S. autonoma* Povolný, 1969 (alt. 3727 m) and *S. dalibori* Lvovsky & Piskunov, 1989 (alt. 2067–2656 m). The habitats in this region are mostly montane grasslands or high mountain deserts, indicating that some species possess strong cold tolerance and high-altitude adaptability.

In summary, conducting taxonomic research on the genus *Scrobipalpa* in Xinjiang enriches the species diversity of the genus in both China and the world, and provides basic morphological and distributional data for systematic classification, revision, and phylogenetic studies of the genus. A small number of species of *Scrobipalpa* feed on plants of Chenopodiaceae and Solanaceae, and some of them may have agricultural or ecological indicator value. As an important agricultural and ecological barrier in China’s arid regions, Xinjiang benefits from systematic taxonomic research on this group, which contributes to future pest monitoring and the conservation of desert ecosystems. The female of *S. apicidentata* sp. nov. remains unknown; future collecting in the type locality (Pishan and Yecheng counties) is needed to complete its morphological characterization. An increase in the number of new species in Xinjiang can be expected both due to the discovery of species known from the adjacent regions, and due to the discovery of undescribed species in the course of further research. Therefore, further collection and investigation in various regions will still be the focus of future research on the genus *Scrobipalpa*.

## Data Availability

The original contributions presented in this study are included in the article. Further inquiries can be directed to the corresponding authors.

## Abbreviations

The following abbreviations are used in this manuscript:

CIS	Centre for Insect Systematics, Kangweon National University, Chuncheon, Korea.
SDEI	Senckenberg German Entomological Institute, Eberswalde, Germany (SDEI formely name as DEI).
HNHM	Hungarian Natural History Museum, Budapest, Hungary.
KSU	Insect Collection of Kashi University, Xinjiang, China.
MfN	Museum für Naturkunde, Universität Humboldt, Berlin, Germany (ZMB or ZMHU formerly name as MfN).
MINGA	Muzeul National de Istorie Naturala Grigore Antipa, Bucharest, Romania.
NHM	Naturhistorisches Museum, Bern, Switzerland.
NHMUK	Natural History Museum, Department of Zoology, London, UK (NHMUK formerly name as BMNH).
NHMW	Naturhistorisches Museum, Wien, Austria.
NKU	Insect Collection, College of Life Sciences, Nankai University, Tianjin, China.
SMNK	Staatliches Museum für Naturkunde, Karlsruhe, Germany (LNK formerly name as SMNK).
TD	Type depository.
TJNHM	Insect Collection of Tianjin Natural History Museum, Tianjin, China.
TL	Type locality.
TLMF	Tiroler Landesmuseum Ferdinandeum, Hall in Tirol, Austria.
DNMNH	Ditsong National Museum of Natural History, Pretoria, South Africa (DNMNH formerly name as TMSA).
UZMH	Zoological Museum, Finnish Museum of Natural History, University of Helsinki, Helsinski, Finland.
ZIN	Zoological Institute, Russian Academy of Sciences, Saint-Petersburg, Russia (ZIN formerly name as ZIRAS).
ZMUC	Zoologisk Museum, Natural History Museum of Denmark, Copenhagen, Denmark.
